# Optimal self-calibration of tomographic reconstruction parameters in whole-body small animal optoacoustic imaging

**DOI:** 10.1016/j.pacs.2014.09.002

**Published:** 2014-09-10

**Authors:** Subhamoy Mandal, Elena Nasonova, Xosé Luís Deán-Ben, Daniel Razansky

**Affiliations:** a Institute for Biological and Medical Imaging, Helmholtz Zentrum München, Neuherberg, Germany; b Faculty of Medicine and Faculty of Electrical Engineering and Information Technology, Technische Universität München, Munich, Germany; c iThera Medical GmbH, Munich, Germany

**Keywords:** Optoacoustic imaging, Speed of sound, Focus measures, Image reconstruction, *In vivo* imaging, Image processing

## Abstract

In tomographic optoacoustic imaging, multiple parameters related to both light and ultrasound propagation characteristics of the medium need to be adequately selected in order to accurately recover maps of local optical absorbance. Speed of sound in the imaged object and surrounding medium is a key parameter conventionally assumed to be uniform. Mismatch between the actual and predicted speed of sound values may lead to image distortions but can be mitigated by manual or automatic optimization based on metrics of image sharpness. Although some simple approaches based on metrics of image sharpness may readily mitigate distortions in the presence of highly contrasting and sharp image features, they may not provide an adequate performance for smooth signal variations as commonly present in realistic whole-body optoacoustic images from small animals. Thus, three new hybrid methods are suggested in this work, which are shown to outperform well-established autofocusing algorithms in mouse experiments *in vivo*.

## 1 Introduction

Optoacoustics offers unique *in vivo* imaging capabilities for preclinical research [1]. However, achieving optimal resolution and contrast as well as associated quality measures in optoacoustic tomographic images implies accurate calibration of the reconstruction parameters. The position and orientation of the ultrasound sensors, spatial variations of the speed of sound (SoS), attenuation and other acoustic properties of the propagation medium may all significantly affect the collected optoacoustic responses [2] and therefore must be correctly accounted for in the image reconstruction process. For example, cross-sectional optoacoustic systems based on single-element [3], [4] or arrays of cylindrically focused transducers [5], [6] are commonly employed due to important advantages derived from reducing the optoacoustic problem into two dimensions. For accurate tomographic reconstructions, the location of all detection points in the imaging plane needs to be precisely known or determined experimentally, the latter by, *e.g.*, imaging a calibration phantom having a uniform and known SoS. Once the acquisition geometry is properly calibrated, the correct values of the acoustic propagation parameters must still be taken into consideration, ideally with the use of an algorithm accounting for acoustic heterogeneities [7], [8], [9], [10], [11]. In many practical cases, the map of SoS variations in the imaged medium is not available a priori nor can be extracted experimentally so representative reconstructions are obtained by considering a uniform heuristically fitted SoS [12], [13].

Dependence of SoS on the temperature of the surrounding matching medium is yet another uncertainty that must be accounted for, *e.g.* by continuously monitoring, the temperature throughout duration of the experiment [14]. Indeed, even subtle temperature variations lead to substantial changes of SoS in water of 2.6 m/s/°C [15]. Consequently, if the water temperature cannot be properly controlled during a prolonged experiment, dynamic calibration of the SoS becomes essential. In addition, local discrepancies between sound propagation velocity in the water and the imaged sample, even under assumption of uniform acoustic properties, may raise the necessity in additional SoS calibration on a per-slice basis. Moreover, fast automatic calibration of the SoS is of high importance in real-time imaging systems, where GPU-accelerated reconstruction algorithms now allow for real-time optoacoustic visualization of the sample in the course of the experiment [16].

Determining autofocusing (AF) parameters for biological images has been a wide area of research and diverse families of methods have been reported for digital microscopy [17], [18], [19], shape from focus [20] and cytogenetic analysis [21]. Some simple AF approaches based on sharpness metrics [22] may perform equally well for optoacoustics, especially when high frequency strongly contrasting image features such as high resolution subcutaneous are present in the images. However, they may not provide an adequately robust performance for smooth or ultrawideband signal variations as commonly present in realistic whole-body optoacoustic images from small animals, especially when considering quantitative model-based reconstructions that preserve low-frequency information [23].

In this work, we discuss on the performance of a number of different AF algorithms for automatic SoS calibration in cross-sectional optoacoustic tomography. Along with investigating a number of measures extensively reported in the literature, we propose additional efficient hybrid focusing metrics employing pre-processing to enhance the focusing performance. The proposed methods further incorporate key improvements, *viz.* edge detection and diffusion, making them optimal for application in optoacoustic SoS self-calibration.

## 2 Materials and methods

### 2.1 Autofocusing algorithms

**Fig. 1:**
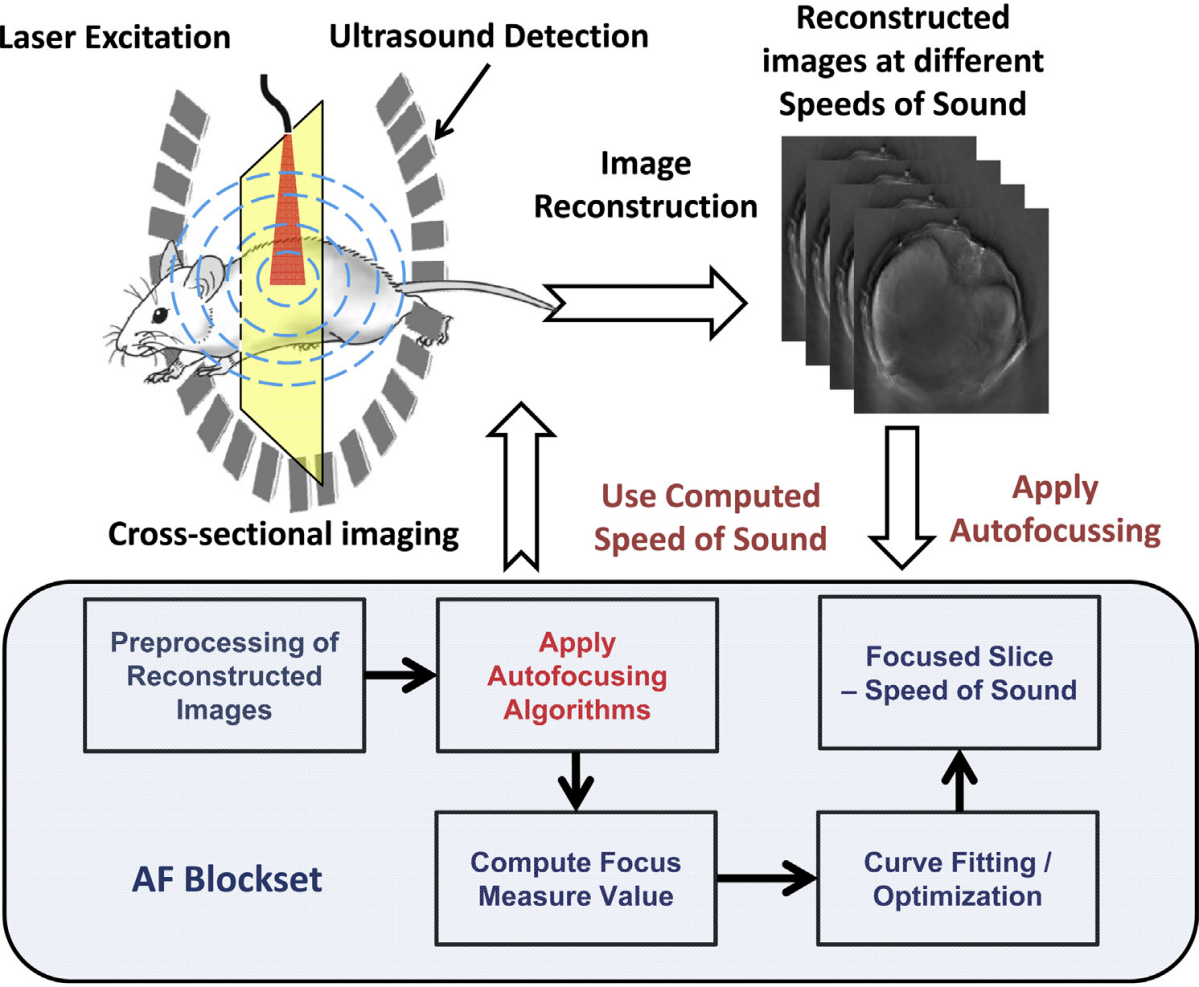
Basic principle of the application of the autofocusing in the optoacoustic reconstruction workflow. The autofocusing (AF) blockset illustrates the post-reconstruction autofocusing algorithm employed to automatically calibrate speed of sound.

The workflow for a typical SoS calibration procedure is depicted in Fig. 1. Optoacoustic images corresponding to selection of different values of the SoS in a certain reasonable range are tomographically reconstructed from the recorded signals. Thereafter, the reconstructed images are processed with the AF algorithm and focus measures are employed to determine the best matching SoS. The fitted SoS, as obtained from the calibration method, is then fed back as a parameter for the reconstruction of the dataset/frame. The algorithms described in this section can be classified into three main groups, namely intensity-based (i and ii), gradient-based (iii and iv) and edge-based (v–vii) measures, where the last group of metrics simultaneously correspond to the hybrid approaches suggested in this work. In order to enable comparison between the different methods, all focus measures are readjusted so that the global minima represent the most focused image. The focus measure is normalized to the maximum value in the SoS range considered. Focus metrics were calculated on the interval from 1460 to 1580 m/s, corresponding to a typical range of SoS in water and soft tissues, with step size of 1 m/s, and processed with smoothing Savitzky–Golay denoising filter (with polynomial order of 0 and window size of 5 points) [24]. The algorithms tested are presented below.

#### 2.1.1 Maximum pixel intensity

The maximum pixel intensity represents the most intuitive and computationally efficient focus measure. The method is inspired by the tendency of the user to look for the brightest spots in the focused image as well as the largest image contrast so that it is assumed that a given structure has the highest intensity value when it is focused. As such, this metric is expected to perform better with high signal-to-noise-ratio (SNR) images rich with high-contrast features, but is the most artifact-prone if noise and other image artifacts yield these high-intensity features. The focus measure is defined as

(1) $$F_{MI}=-\max_{x,y}[f(x,y)]\text{,}$$

where *f*(*x*,*y*) is a function of two variables representing the gray level intensity in the cross-sectional image. The negative sign is added so that the global minimum represents the most focused image, as mentioned above.

#### 2.1.2 Maximum intensity range

The maximum intensity range is a modified version of the previous method [18]. In this case, the difference between the maximum and minimum pixel intensity is calculated, *i.e.*, the focus measure is defined as

(2) $$F_{MIR}=-\{\max_{x,y}[f(x,y)]-\min_{x,y}[f(x,y)]\}\text{.}$$

#### 2.1.3 Brenner's gradient

The Brenner's gradient provides a quantitative measure of image sharpness. It is based on computing the difference between the intensity values for pixels separated by two times the pixel size. In two dimensions, it can be expressed as

(3) $$F_{BG}=-\{\Sigma_{x,y}{\left[f(x+2,y)-f(x,y)\right]}^{2}+\Sigma_{x,y}{\left[f(x,y+2)-f(x,y)\right]}^{2}\}\text{.}$$

The Brenner's gradient is a widely used metric and it has been shown to outperform other methods for SoS calibration in three-dimensional optoacoustic imaging [22].

#### 2.1.4 Tenenbaum's gradient

The Tenenbaum's gradient uses an edge-detection-based approach (sharper edges correspond to higher frequencies). The gradient is determined by a convolution between the Sobel operator (and its transpose) with the image pixels. This focus measure is calculated as

(4) $$F_{TG}=-\{\Sigma_{x,y}{\left[G*f(x,y)\right]}^{2}+{\left[G^{T}*f(x,y)\right]}^{2}\}\text{,}$$

where

(5) $$G=\left[\begin{array}{ccc} -1 & 0 & +1\\ -2 & 0 & +2\\ -1 & 0 & +1 \end{array}\right]$$

represents the Sobel operator and * denotes two-dimensional convolution. While the Tenenbaum's gradient has been reported to be superior in microscopy [25], its performance in optoacoustic imaging has been shown to be comparable to that of the Brenner's gradient [22].

#### 2.1.5 Normalized sum of edge pixels (Edge + Sum)

The normalized sum of edge pixels calculates the sum of pixels corresponding to strong edges, subsequently normalized by the total number of pixels in the image. The Sobel approximation to the derivative is used as edge detection algorithm. This metric aims at minimizing the influence of thin circles and ‘crossing-arcs’ artifacts typically present in unfocused cross-sectional optoacoustic images. The method then aims at maximizing clearly defined edges, *i.e.*, it represents, to some extent, an opposite approach to the traditional camera focusing. The focus measure is then expressed as

(6) $$F_{ES}=\frac{1}{N}\Sigma_{x,y}e(x,y)\text{,}$$

being *N* the number of pixels in the image and

(7) $$e(x,y)=\left\{\begin{array}{ll} 1, & g(x,y)>\text{threshold}\\ 0, & \text{otherwise} \end{array},\right)$$

with

(8) $$g(x,y)=\sqrt{{\left[G*f(x,y)\right]}^{2}+{\left[(G^{T})*f(x,y)\right]}^{2}}\text{.}$$

The value of the threshold was determined automatically by computing the root mean squared (RMS) estimate of noise [26].

#### 2.1.6 Normalized variance of the image gradient magnitude using Sobel operator (Sobel + Var)

The normalized variance of the image itself has been previously reported as focus measure in computer microscopy [17], [27] and later in optoacoustic imaging [22]. Herein, we suggest an additional step consisting in computing the variance of the gradient magnitude obtained by convolution with the Sobel operator. This metric belongs to hybrid approaches being combination of statistics-based and derivative-based algorithms, leading to an enhanced performance in optoacoustic images having a relatively low contrast compared to natural images. The focus measure is expressed as

(9) $$F_{SV}=-\frac{1}{N\mu }\Sigma_{x,y}{\left(g(x,y)-\mu \right)}^{2}\text{,}$$

where μ is the mean value of *g*(*x*,*y*), as defined in (8).

#### 2.1.7 Anisotropic diffusion enhanced energy of image gradient using consistent gradient operator (Ad-CG)

Another hybrid methodology based on a combination of anisotropic diffusion and consistent gradient (CG) operator is suggested in this work. Anisotropic diffusion is an iterative scale-space approach, which enhances the edges while smoothing the rest of the information in the image [28]. The purpose of this pre-processing step is twofold, namely, to remove noise and intensity fluctuations on the one hand, on the other – to reduce the ripples in focus measure as a function of the SoS. The continuous form of the non-linear partial differential equation (PDE) as proposed by Perona and Malik [28] for diffusing an image is given by [29]

(10) $$\left\{\begin{array}{l} \frac{\partial I}{\partial t}=div[C(|\nabla I|)\cdot \nabla I]\\ I(t=0)=f_{0}(x,y) \end{array}\right)$$

where ∇ and *div* are the gradient and divergence operators, respectively, *C*(*x*) is the diffusion coefficient, and *f*_0_(*x*,*y*) is the initial image. Eq. (10) is solved iteratively as explained in [28], where the diffusion coefficients is taken as

(11) $$C(x)=\frac{1}{1+{\left(x/k\right)}^{2}}\text{,}$$

with *k* being an edge magnitude parameter.

A consistent gradient (CG) operator is then applied as a second step to compute the energy of the image gradient which is translated as focus measure scores [30]. This approach has been reported to have more stable AF performance under varying illumination conditions for microscopic imaging [31], [32]. The use of a CG operator ensures the exactness of gradient direction in a local one-dimensional pattern irrespective of orientation, spectral composition, and sub-pixel translation. The energy of the image gradient is defined as:

(12) $$E=\int_{-\infty }^{\infty }\int_{-\infty }^{\infty }|\nabla f(x,y){|}^{2}dx\;dy$$

The actual focus measure including all intermediate steps can be expressed in a form:

(13) $$F_{ADCG}=\frac{1}{N}\sum_{x,y}w\cdot I_{H}+(1-w)\cdot I_{V}\text{,}$$

where *N* is the total number of image pixels and *w* is an additional factor allowing for flexibility in assigning more weight to horizontal *I*_*H*_ or vertical *I*_*V*_ derivative approximations defined as

(14) $$I_{H}=CG*I_{AD};\;I_{V}=CG^{T}*I_{AD}\text{,}$$

being *I*_AD_ the optoacoustic image after anisotropic diffusion filtering and CG the 5 × 5 consistent gradient operator expressed as [30], [32]

(15) $$CG=\left[\begin{array}{ccccc} -0.003776 & -0.010199 & 0 & 0.010199 & 0.003776\\ -0.026786 & -0.070844 & 0 & 0.070844 & 0.026786\\ -0.046548 & -0.122572 & 0 & 0.122572 & 0.046548\\ -0.026786 & -0.070844 & 0 & 0.070844 & 0.026786\\ -0.003776 & -0.010199 & 0 & 0.010199 & 0.003776 \end{array}\right]\text{.}$$

### 2.2 Experimental setup

The experimental performances of the AF algorithms were tested in cross-sectional optoacoustic acquisition geometry [5] using a commercial small animal multispectral optoacoustic tomography (MSOT) scanner (Model: MSOT256-TF, iThera Medical GmbH, Munich, Germany). In short, the scanner consists of a custom-made 256-element array of cylindrically focused piezocomposite transducers with 5 MHz central frequency for simultaneous acquisition of the signals generated with each laser pulse. The transducer array covers an angle of approximately 270° and has a radius of curvature of 40 mm. Light excitation is provided with the output laser beam from a wavelength-tunable optical parametric oscillator (OPO)-based laser, which is shaped to attain ring-type uniform illumination on the surface of the phantoms by means of a custom-made fiber bundle. The detected optoacoustic signals are simultaneously digitized at 40 MS/s. The scanner is capable of rendering 10 cross-sectional images per second but here the images were averaged 10 times in order to improve SNR performance in acquiring entire mouse cross-sections.

### 2.3 Image reconstruction

The acquired signals were initially band-pass filtered with cut-off frequencies between 0.1 and 7 MHz for removing low frequency offsets and high frequency noise, and subsequently input to a reconstruction algorithm rendering a cross-sectional distribution of the optical absorption. Two alternative image formation approaches were considered, namely, the back-projection reconstruction and model-based inversion. The former one is based on a delay-and-sum approach [33], [34], whereas several back-projection formulas are available. For a finite number of measuring locations, the optical energy deposition $f({x^{\prime }}_{j},{y^{\prime }}_{j})$ at a given pixel of the region of interest (ROI) was calculated via [16]

(16) $$f({x^{\prime }}_{j},{y^{\prime }}_{j})=\sum_{i}s(x_{i},y_{i},t_{ij})\text{,}$$

where (*x*_*i*_,*y*_*i*_) is the *i*^th^ measuring location and $t_{ij}=|({x^{\prime }}_{j},{y^{\prime }}_{j})-(x_{i},y_{i})|/c_{o}$, being *c*_*o*_ the SoS. *s*(*x*_*i*_,*y*_*i*_,*t*_*ij*_) represents the function to be back-projected, which in our case was taken as the filtered pressure. Even though the approximated closed-form back-projection algorithms may lead to fast (real-time) and qualitatively good-looking images, they have been shown to artificially accentuate high frequency image features, provide negative image values, and further result in other substantial image artifacts, in particular in limited view optoacoustic tomographic geometries [35], thus hindering their efficient implementation for quantitative image reconstruction.

In addition, performance of the different focusing methods for images reconstructed with the exact numerical model-based reconstruction algorithm, termed interpolated-matrix-model inversion IMMI [23], was further investigated. This class of algorithms has been generally shown to retain the quantitative nature of optoacoustic reconstructions by taking into account the various experimental imperfections [35], [36], better preserving the low frequency information, and mitigating other image artifacts associated with the approximated back-projection schemes [35]. It is based on a least-squares minimization between the measured pressure at a set of locations and instants (expressed in a vector form as **p**) and the equivalent theoretical pressure predicted by a linear model obtained from a discretization of the optoacoustic forward solution. The optical absorption at the pixels of the ROI, expressed as vector form **F**, is calculated as follows

(17) $$\text{F}=\arg\min_{\text{f}}||\text{Af}-\text{p}|{|}^{2}+\lambda^{2}||\text{Lf}|{|}^{2}\text{,}$$

where **A** is the linear operator (or model matrix) mapping the optical absorption to the acoustic pressure. Details on how to calculate the matrix **A** are provided in [10]. Standard Tikhonov regularization was employed to minimize the high-frequency noise in the inversion process, which is particularly beneficial in presence of limited view problems. The matrix **L** represents a high-pass filter operation as described in [10]. The model-based inversion procedure can be further modified to account for speed of sound variations in the medium [10] or to minimize the artifacts due to internal reflections [37] in case these effects cause undesired distortion in the images. In the current implementation, the computational time of back-projection and model-based reconstructions for generating a stack of 100 images at different SoS (200 × 200 pixels) are approximately 8.818 s and 888.442 s, respectively. Workstation with Intel i7-480 CPU operating at 3.70 GHz and with 32 GB of RAM is used for the experimentation. The back- projection reconstruction is further accelerated using the OpenGL platform on AMD Redeon GPU (Clock speed- 1100 Mhz, Memory size 3072 MB, Shaders 2048).

Both back-projection and model-based algorithms are initially derived for a propagation medium with a uniform SoS. Although both algorithms can be potentially modified for heterogeneous acoustic media at the cost of algorithmic and computational complexity [10], [13], a reasonable-quality image can often be rendered using (16), (17) even if small variations of the SoS are present in the sample [13]. In the experiments performed herein, a grid of 200 × 200 pixels corresponding to a field of view of 25 mm × 25 mm (125 μm pixel size) was employed, which is adapted to the actual resolution of the system [5].

### 2.4 The imaging protocol

Two experiments were conducted in order to test the AF algorithms. In a first experiment, a murine kidney (excised post-mortem) embedded in an agar phantom was imaged *ex vivo*. The phantom was made with an agar solution (1.3% agar powder by weight) containing 1.2% by volume of Intralipid to provide uniform light fluence at the kidney surface. In the second experiment, 30 imaging datasets corresponding to 10 different mice were acquired *in vivo*. The mouse datasets were drawn from three regions of anatomical significance, namely the brain, liver and kidney/spleen regions. The wavelength of the laser was set to 800 nm in all experiments and the water temperature was maintained at 34 °C.

## 3 Results

**Fig. 2:**
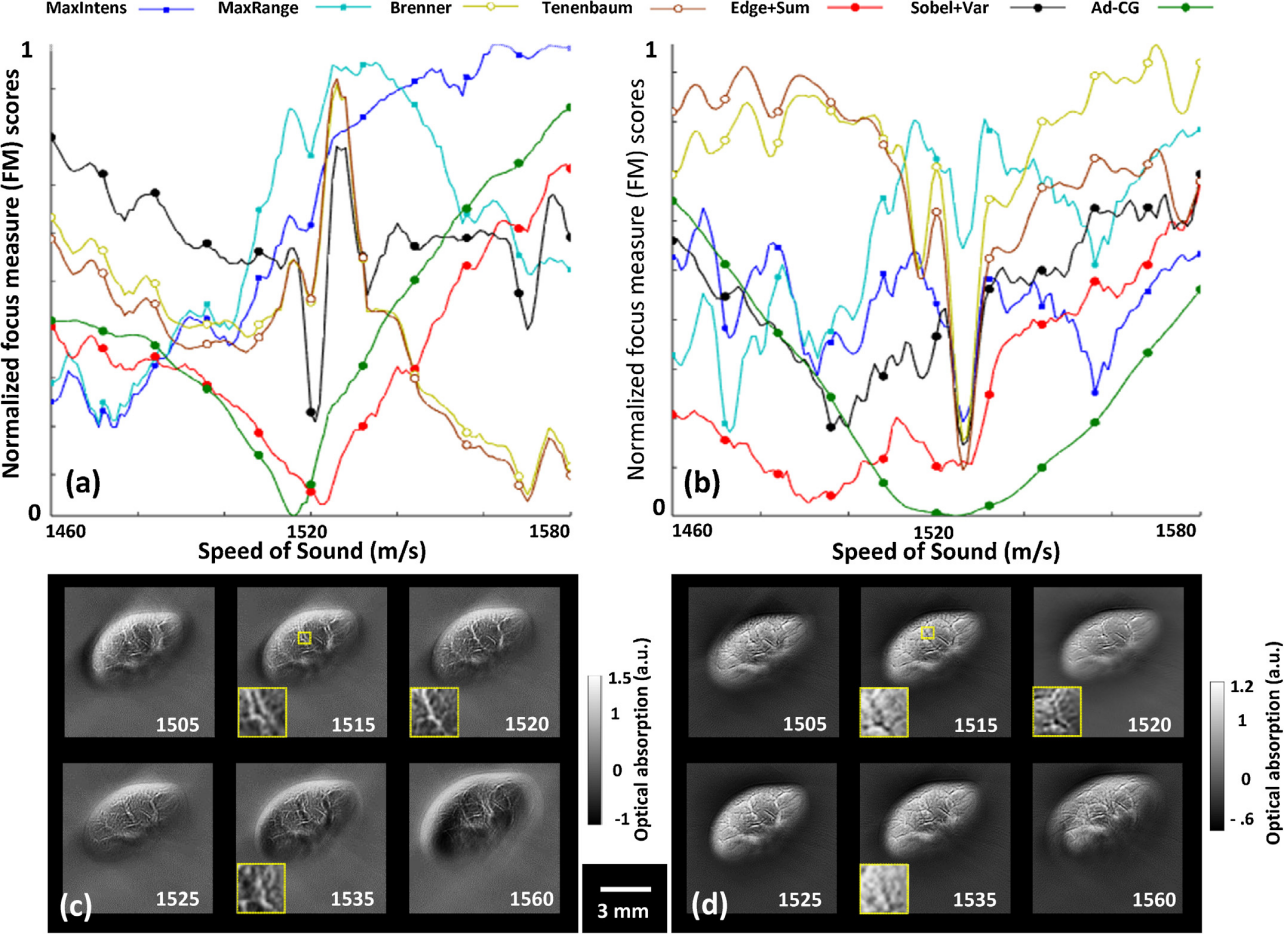
Speed of sound calibration for an *ex vivo* organ (murine kidney). The graphs show the normalized focus measures versus the speed of sound for 7 different focus measures using (a) back-projection and (b) model-based reconstruction methods. For all focus measures the global minima determine the most focused image. Panels (c) and (d) show the images at six different speeds of sound reconstructed with back-projection and model-based algorithms, respectively (values are stated in [m/s]). A zoom-in of a representative region inside the object is showcased for a better visual evaluation of the image quality enhancement achieved with the proper value of the speed of sound.

Results for the *ex vivo* murine kidney experiment are displayed in Fig. 2. In an effort to test whether the reconstruction methodologies have an impact on the outcome of the calibration procedure, back-projection and model-based inversion methods were compared in this case. Even though all images were manually thresholded to attain best visual appearance, the image quality is generally improved with the model-based approach over the back-projection reconstructions, the latter exhibiting generally unreasonable distribution of the optical absorption with pronounced negative value artifacts across the imaged sample. The calculated focus measures as a function of the SoS are showcased in Fig. 2a and b for back-projection and model-based reconstruction, respectively. All focus measures are normalized to the maximum value in the SoS range. A Savitzky–Golay denoising filter was further applied as a valuable additional step for removing spikes from the focus measure plots, thus avoid ambiguity and locking up into local minima.

The focus measure is expected to have a minimum for the value of the SoS corresponding to the best focused image. Indeed, most of the metrics reach the same calibration SoS regardless of the reconstruction method. Examples of reconstructed images with back-projection and model-based reconstruction of the *ex vivo* murine kidney for several equally spaced SoS values are displayed in Fig. 2c and d respectively, where the subjectively best-looking images correspond approximately to the minimum of most focus measures. The metrics generally show sharper focusing performance with back-projection reconstruction, probably due to higher frame-to-frame variability when the SoS was changed. On the other hand, the focus scores were generally more consistent for the model-based reconstructions. The focusing curves were less noisy for the Ad-CG method, where 2 iterations were used in the anisotropic diffusion step, as determined empirically.

**Fig. 3:**
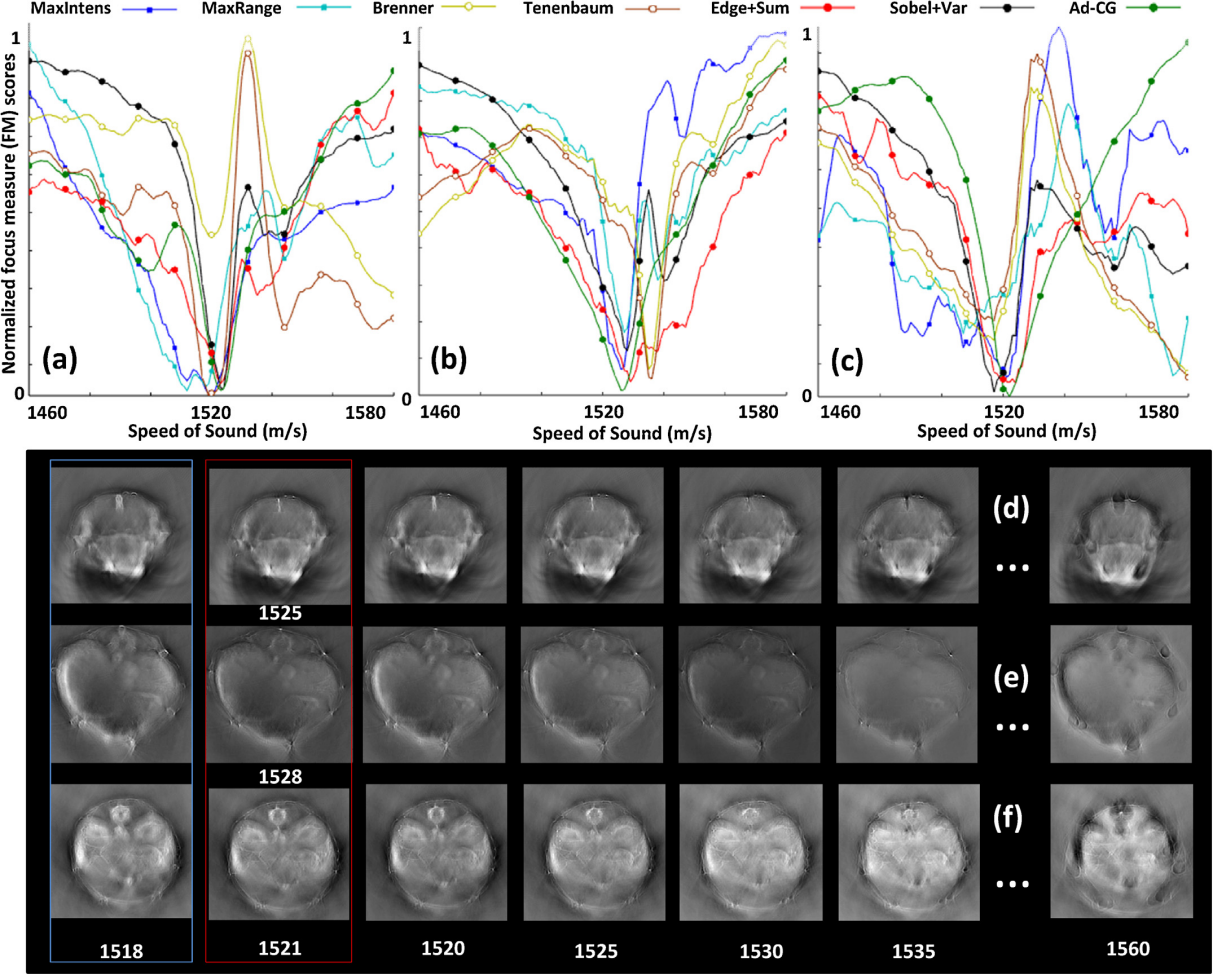
Focus measure (FM) plots for 7 different metrics in three different anatomical regions of the mouse during *in vivo* imaging of (a) brain, (b) liver, and (c) kidney/spleen. The global minima of the focus measure score represent the calibrated speed of sound. Reconstructed images at different speed of sound values for the respective regions are shown in (d–f), where the first and second columns correspond, respectively, to the speed of sound in water (at 34 °C) and the speed of sound manually fitted.

Fig. 3 displays the *in vivo* mouse imaging results. In particular, the focus measures for the head, liver and kidney/spleen regions as a function of the SoS are showcased in Fig. 3a–c, respectively. Representative images for these three regions of the mouse body obtained by considering different values of the SoS are accordingly shown in Fig. 3d–f. All images were reconstructed with the back-projection approach. The numbers of iterations in the anisotropic diffusion procedure were heuristically chosen as 4, 12 and 18 for the liver, brain and kidney/spleen regions, respectively. The iterations ensures that a sufficient level of smoothening is achieved without blurring edges, thus different number of iterations were determined for each region imaged based on observation and inherent nature of the images. Further, a fixed edge weight ‘*w*’ (see Eq. (13)) of 0.95 was used for all the experiments with Ad-CG method. The choice of number of iteration is thus critical for the good performance of the algorithm, effects of weighting is limited for the current modality but might have greater applicability in the presence of strong limited view problems.

A higher variability in the focus measures was noticed for the brain images, primarily due to the lack of well-defined structures and edges to focus on. For example, the Tenenbaum measure yielded a minimum at the upper limit of the SoS range, where the reconstructed image for this particular selection (Fig. 3d) is clearly deteriorated. The performance of the metrics was better for the kidney/spleen and the liver regions given higher intrinsic contrast and defined vascular structures found in these areas. As a first approach, the temperature of the coupling medium (water) can be used for referencing the SoS and using it for reconstructing the data. However, as clearly shown in Fig. 3d–f (first column), the resulting images obtained for this value of the SoS are not optimal. Indeed, the average SoS in soft tissues is approximately 1540 m/s [38], and can have variations of up to 10% with respect to the SoS in water [15]. A different (generally higher) SoS must then be used for the reconstruction, and the AF algorithms provide a suitable platform for this purpose. The manually selected values of the SoS are highlighted in the second column of Fig. 3. The manual calibration values were decided based on subjective testing using feedback from three independent volunteers experienced in reading animal anatomy but with no prior knowledge of the SoS calibration values. SoS retrieved with the proposed AF algorithms fits best the one selected manually using the subjective testing. It is worth noticing that only the three last hybrid focus metrics, especially the Ad-CG, have the sharp peak on the entire interval, probably due to the diffusing (or smoothing) processing of the image. This in turn minimizes the chances for secondary local minima to appear, which may lead to misinterpretation of the results. It is to be noted that, anisotropic diffusion is a well-known image processing technique that successfully reduces image noise without compromising significant parts of the image content, typically edges, lines or other details that are essential for image interpretation and analysis [28].

**Fig. 4:**
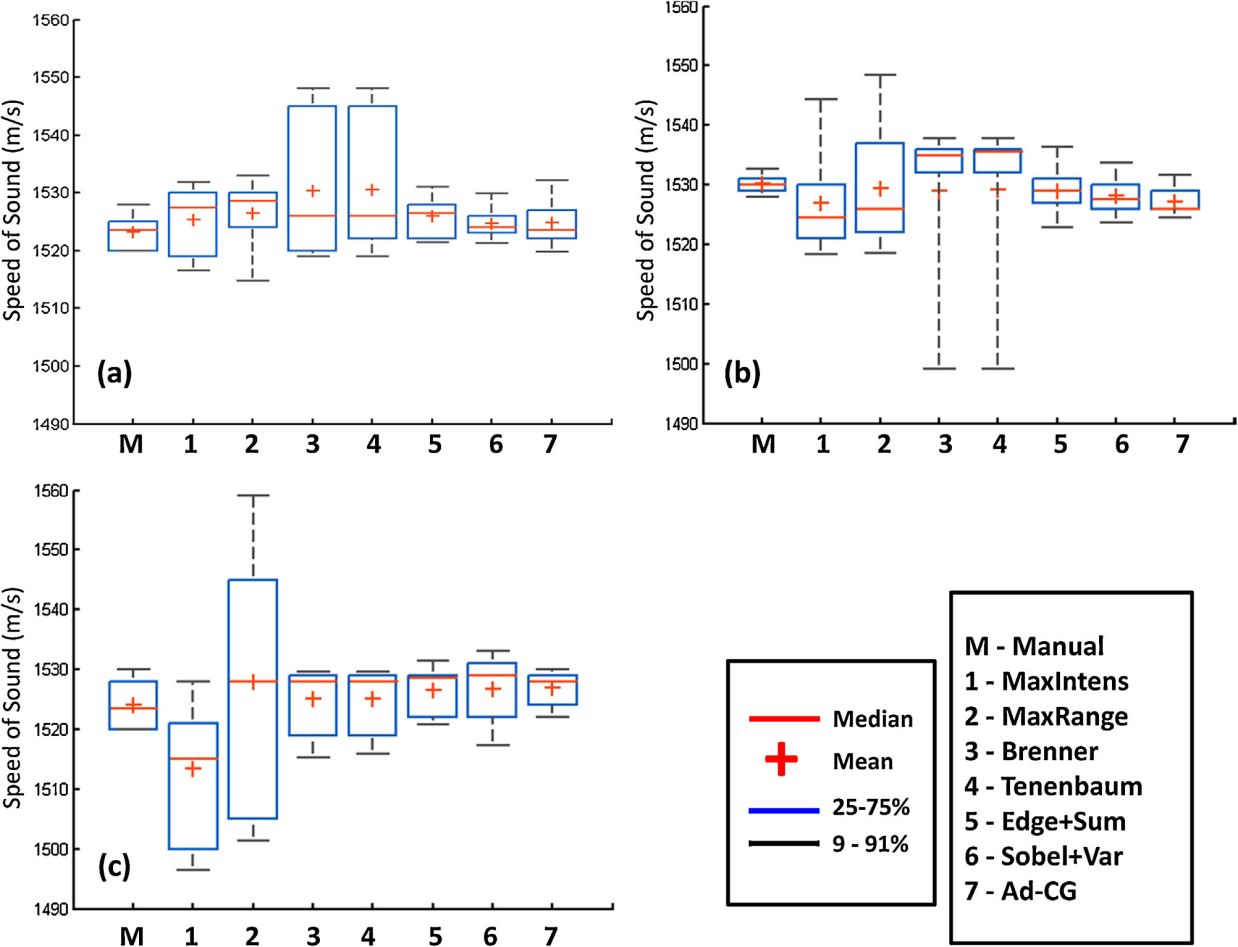
Boxplots indicating the speed of sound variability for 10 independent datasets for (a) brain, (b) liver, and (c) kidney/spleen regions. User feedback was taken for the manual calibration and the 7 automated metrics were compared against it.

To quantify the overall efficacy of the results, tests on 10 datasets for each of the designated regions in mice were conducted. The boxplots of the resulting values of the SoS are shown in Fig. 4. The gradient-based methods, *i.e.* Brenner's and Tenenbaum's gradients, generally performed satisfactorily, in agreement with earlier publications [22], [39], although secondary drifting peaks often appear, which severely offset the global minima value. The effects of such secondary fluctuations were reduced with the filtering process and by considering only a SoS range between 1480 and 1560 m/s. Secondary peaks also appeared in some cases when considering the Edge + Sum algorithm, although the resulting variability is lower. On the other hand, the performance of the two proposed metrics Sobel + Var and Ad-CG are consistent (no secondary peaks appeared) and provide variability similar to that obtained by manual selection. The worst performance in terms of variability and fitted SoS value have been obtained by the intensity-based methods, presumably due to the highest susceptibility to noise and artifacts.

## 4 Discussion and conclusions

The applicability of focusing techniques for automatic calibration of a uniform speed of sound value in optoacoustic tomographic reconstructions has been analyzed in this work. For the particular implementation in cross-sectional whole body optoacoustic small animal imaging, efficacy of two of the suggested methods, namely, the normalized variance of the image gradient magnitude using Sobel operator and the algorithm employing anisotropic-diffusion-enhanced energy of the image gradient using consistent gradient operator, was found superior to the other established focus measures.

The need for autofocusing in optoacoustic tomographic imaging stems from the fact that the average SoS in the region covered by the measuring locations is unknown. Even if the geometrical distribution of the tomographic detection points is accurately calibrated and the water temperature is known, the corresponding SoS in water for such temperature generally does not lead to the optimum results. This effect has been illustrated in this work, where the self-calibrated SoS was generally higher than the SoS in water. This result is consistent with the fact that the average SoS in soft tissues is slightly higher than that in water, with variations reaching up to 10% [15]. The SoS in water can, however, be used as an initial guess that may serve as the central SoS of the search interval and thus ease the optimization of the focus measures. On the other hand, although representative images can be rendered with algorithms assuming a uniform SoS, more accurate reconstructions may require considering a heterogeneous distribution, and AF may also play a similar role in fitting the SoS of defined regions.

A good performance of the methods analyzed in this work has been demonstrated for images reconstructed with two different reconstruction algorithms. Indeed, whereas back-projection reconstruction highlights the high spatial frequency components of the image, model-based inversion generally renders more quantitative images by accurate estimation of the low-frequency background. On the other hand, the computational burden for back-projection reconstructions is usually significantly lower [40] so this approach is more convenient for fast (dynamic) calibration of the SoS during real-time operation. Essentially, the best performing metrics would not only show good performance in phantoms or imaging of subcutaneous vasculature but also in cases of *in vivo* imaging of entire animal cross-sections. A good performance was obtained in mouse experiments *in vivo* for three representative regions corresponding to the location of the brain, liver and kidney/spleen. However, secondary peaks in the focus measures led in some cases to erroneous interpretations, which increase variability of the results. The best results in terms of consistency (as compared with manual fitting) and low variability were achieved with the hybrid approaches suggested in this work.

Even though the current paper only showcases self-calibration in the case of SoS, autofocusing approaches may readily find broader applicability in calibrating other parameters in optoacoustic tomographic imaging systems. For instance, the position and orientation of ultrasound sensors is generally unknown, especially in self-developed systems, and must be calibrated in a first place. Selecting the most focused plane in the elevation direction around certain structures may also represent a potential application in the case of cross-sectional (two-dimensional) imaging systems. Finally, the behavior of the methods in three-dimensional optoacoustic imaging needs to be further analyzed [41], [42].

In conclusion, similarly to optical microscopy techniques, focusing techniques are expected to play a fundamental role in the calibration of optoacoustic reconstruction parameters, particularly the SoS. The showcased performance of the suggested methods in cross-sectional imaging systems anticipates their general applicability for preclinical and clinical imaging with other geometrical configurations. Furthermore, the self-calibration of reconstruction parameters allows one to reliably reconstruct large datasets of whole animal imaging with minimal operator intervention – thus effectively addressing the problems of processing larger volumes of data, especially as optoacoustics progresses toward high throughput biological imaging applications.

## Conflict of interest

The authors declare that there are no conflicts of interest.
